# Systematic overdosing of oxa- and cloxacillin in severe infections treated in ICU: risk factors and side effects

**DOI:** 10.1186/s13613-017-0255-8

**Published:** 2017-03-22

**Authors:** Mathilde Neuville, Najoua El-Helali, Eric Magalhaes, Aguila Radjou, Roland Smonig, Jean-François Soubirou, Guillaume Voiriot, Alban Le Monnier, Stéphane Ruckly, Lila Bouadma, Romain Sonneville, Jean-François Timsit, Bruno Mourvillier

**Affiliations:** 1 Service de Réanimation médicale et Infectieuse, Hôpital Bichat-Claude Bernard, Assistance Publique-Hôpitaux de Paris, 46 rue Henri Huchard, 75018 Paris, France; 2 Unité de Microbiologie clinique et dosages des anti-infectieux, Groupe Hospitalier Paris Saint-Joseph, 185 rue Raymond Losserand, 75014 Paris, France; 3 UMR 1137 - IAME Team 5 – DeSCID: Decision Sciences in Infectious Diseases, Control and Care, INSERM, Paris Diderot University, 75018 Paris, France

**Keywords:** Cloxacillin, Intensive care, Side effects, Endocarditis, Renal failure

## Abstract

**Background:**

Oxacillin and cloxacillin are the most frequently used penicillins for the treatment of severe methicillin-susceptible *Staphylococcus aureus* infections in intensive care units (ICUs), especially endocarditis. International recommendations do not suggest any adaptation of the dosage in case of renal impairment. We wanted to assess the risk factors for overdosing in ICU and the related observed side effects.

**Methods:**

All patients with a therapeutic drug monitoring of oxa- or cloxacillin between 2008 and 2014 were included. The target range of trough concentration for total antibiotic activity was considered to be 20–50 mg/L. Data concerning the infection, the given treatment, the renal function, and the attributed side effects of overdosing were collected. A logistic regression model was used to compute the measured trough concentrations.

**Results:**

Sixty-two patients were included in this study. We found a median trough plasma concentration of 134.3 mg/L (IQR 65.3–201 mg/L). Ten patients (16.1%) reached the target concentration; all other patients (83.9%) were overdosed. Eleven patients (17.7%) experienced neurological side effects attributed to a high antibiotic concentration, i.e. persistent coma and delirium. When adjusted on the dosage used, the risk of overdosing was significantly associated with a creatinine clearance <10 mL/min (with or without hemodialysis).

**Conclusion:**

With the suggested dose of 12 g/day for cloxacillin treatment in case of endocarditis and severe infections occurring in ICU, 83.9% of patients are largely overdosed. Considering the observed side effects, doses should be accurately monitored and reduced, particularly when renal replacement therapy is needed.

**Electronic supplementary material:**

The online version of this article (doi:10.1186/s13613-017-0255-8) contains supplementary material, which is available to authorized users.

## Background

Beta-lactams are first-choice antibiotics for most infections in intensive care units [1]. Oxacillin and cloxacillin are the most frequently used penicillins for the treatment of methicillin-susceptible *Staphylococcus aureus* (MSSA) infections. Since this bacterium is responsible for more than one quarter of all endocarditis, in both a nosocomial and community context [2–4], these antibiotics stand at the forefront of the latest European Society of Cardiology guidelines [5]. Given such criteria as safety of use and low level of toxicity, as opposed to the well-known effect of aminoglycosides or vancomycin [6], the therapeutic drug monitoring of these molecules has never been widespread.

As all penicillins, oxacillin and cloxacillin are time-dependent antibiotics. For severe infections, the target is to obtain 100% of time above the minimum inhibitory concentration (MIC), or even 4× MIC to avoid emergence of resistances [7]. To reach this concentration, high doses and prolonged infusion are recommended in ICU patients with an increased distribution volume [8, 9] and glomerular hyperfiltration [10]. The dosage of 12 g/day (200 mg/kg/day as specified only for children) is recommended by consensus conferences to treat severe infections, without mentioning any specific attention in case of renal failure [5, 11]. Recently, the French Health Authority (Haute Autorité de Santé or HAS) has suggested to reduce the dose by 50% when creatinine clearance falls below 30 mL/min [12], leading to subsequent modifications of the medication approval.

There is also a lack of data in the literature concerning a possible accumulation in the event of prolonged use, particularly in case of endocarditis or endovascular infections. Thus, ICU patients are potentially exposed to severe complications, essentially neurological [13, 14], hepatic [15, 16], and renal [17–19].

The aim of our study was to analyze oxacillin and cloxacillin plasma concentrations in critical care patients in order to identify risk factors for overdosing, its frequency, and the observed adverse effects.

## Methods

### Clinical and biological data

All patients with oxacillin or cloxacillin therapeutic monitoring performed between 2008 and 2012 in the intensive care units of Hôpital Bichat, Paris, France, were retrospectively included in the study. Patients from 2012 to 2014 were included prospectively after 72 h of administration of treatment in our ICU. We collected clinical characteristics, medical history, characteristics of the infectious process, date of the infection, date of oxacillin start, and given dose. Creatinine clearance has been systematically measured with the following formula:

$$\frac{{C_{\text{creatinine}} {\text{ in urine}}\left( {\text{ mmol/L}} \right) \times {\text{volume of urine for the last }}24\,{\text{h}}\left( {\text{ mL}} \right) \times 1000}}{{C_{\text{creatinine}} {\text{ in plasma }}\left( {\upmu{\text{ mol/L}}} \right) \times 1440}}$$

When data were missing, glomerular filtration rate was estimated with MDRD formula. We checked that the plasma concentrations matched the trough blood concentration before a new injection and identified the time lag between the plasma concentration measure and the latest hemodialysis session, when appropriate. We also addressed issues relating to hepatic function, serum protein level and serum albumin level when available. We estimated the unbound fraction of antibiotics according to the serum albumin or protein level. Other associated antimicrobials were also recorded, notably aminoglycosides. The day of ICU admission was called Day-O, the beginning of oxa- or cloxacillin treatment T-Day, and the day of plasma concentration measure D-Day. The dosing of antibiotics was reduced in case of renal failure, i.e. when creatinine clearance was inferior to 30 mL/min. Usually, in cases of endocarditis or endovascular infections, 2 g were administered four times a day instead of six, the latter being the recommended dosage for the treatment of endocarditis. Finally, Sequential Organ Failure Assessment (SOFA) score [20] and Simplified Acute Physiology Score (SAPS) II [21] were assessed at the time of admission and drug monitoring. The Naranjo Adverse Drug Reaction Probability Scale was used to assess whether the observed adverse effects were due to the drug or not, via a score termed definite, probable, possible, or doubtful [22].

### Oxacillin and cloxacillin assays

The concentration of cloxacillin or oxacillin in serum was measured by microbiological assay with *Bacillus subtilis* as assay organism, which is resistant to third-generation cephalosporins, macrolides, quinolones, fusidic acid, rifampin, fosfomycin, and cyclines. Standard solutions of different concentrations of the tested antibiotic were prepared. Hundred milligrams of cellulose phosphate was added to patients’ serum in order to adsorb aminoglycosides. After 30 min and a centrifugation, the collected supernatant did not contain aminoglycoside anymore. A volume of 25 microliters of each standard solution containing cloxacillin or oxacillin and patient’s sample were poured on sterile filter papers of 6 mm placed on plates made with a *Bacillus subtilis* broth. Each measure was repeated three times. The plates were refrigerated at 4 °C to facilitate the diffusion of the antibiotic and then incubated at 37 °C for 18 h. The inhibition zone diameters were measured, and the concentrations of the test specimens were derived from the standard line constructed from the standard solution.

### Pharmacokinetics and pharmacodynamics (PK/PD) target

According to the European Committee on Antimicrobial Susceptibility Testing (EUCAST), the MIC threshold to determine the strain sensitivity is 0.5 mg/L for oxacillin and 2 mg/L for cloxacillin [23]. Until now, the optimal plasma concentration target in order to achieve the best cure rate remains undefined, but as oxa- and cloxacillin are time-dependent antibiotics, a time of 100% above the minimum inhibitory concentration (MIC) was considered to be optimal for efficacy. Considering that oxa- and cloxacillin are highly bound to albumin and that their unbound fraction—i.e. the available fraction for antibiotic efficacy—in healthy patients is known to be 10%, we defined a target range of total antibiotic concentration of 20–50 mg/L to reach the 100% *T* > MIC.

### Estimation of the unbound fraction of antibiotics

Considering that oxa- and cloxacillin are highly bound to albumin (up to 90%) [24], we assumed that the unbound fraction increased in hypoproteinemic patients. In the study by Ulldemolins et al. [25] involving flucloxacillin, the relationship between percentage protein binding and plasma albumin levels was found to be linear, with *r* ^2^ values of 0.5691. We estimated the increase in the unbound fraction with the percentage of decrease in albuminemia, extrapolated from the ratio albumin/serum protein when albumin was not available. In our hospital laboratory, hypoalbuminemia was defined as a value inferior to 32 g/L.

### Statistical analysis

Clinical characteristics and therapeutic drug monitoring were reported as median IQR or n (%) for quantitative and qualitative variable, respectively. Variables associated with a plasma concentration superior to the target range of 20–50 mg/L were computed using a logistic regression model. Antibiotic dose (mg/kg) was forced in the models, providing that the dose was adapted to renal function by the clinician. A *p* value of 0.05 was considered significant. All the statistical analyses were performed using SAS 9.4 and R softwares.

## Results

Sixty-two patients were included in this study, representing 94 drug monitorings. For patients included prospectively after 2012, a flowchart is available in Additional file 1: Figure S1. Cloxacillin was the most frequently used molecule in our work, as it was administered to 56 patients (90.3%), whereas oxacillin was given to the six others (9.7%).

Data concerning the first plasma concentration measure performed for each patient are summarized in Table 1. Twenty-three patients (37% of the cases) were mechanically ventilated with a P/F ratio under 200, 37 (59.7%) had a creatinine clearance of less than 10 mL/min requiring renal replacement therapy for 33 (53%) of them, and 30 (48%) required catecholamines. Data to calculate creatinine clearance were missing for nine patients; with MDRD formula, one of them had an estimated creatinine clearance between 10 and 30 mL/Min, and the eight others above 30 mL/min. The ICU mortality was 41.9% (*n* = 26). For 96% of the patients, the involved bacterium was *Staphylococcus aureus;* two patients suffered from *Coagulase*-*negative Staphylococcus*- and *Staphylococcus epidermidis*-related infections.

**Table 1:** Patients characteristics

Sex, number (%)	
F	16 (25.8)
M	46 (74.2)
Age, years, median (IQR)	63 [54; 71]
Weight at Day 0, kg, median (IQR)	80 [73; 92]
BMI, median (IQR)	27.8 [24.3; 32.5]
Cause of admission, number (%)	
Sepsis	38 (61.3)
Acute respiratory failure	9 (14.5)
Coma	9 (14.5)
Cardiogenic shock	6 (9.7)
Renal replacement therapy	6 (9.7)
Post-surgery	5 (8.1)
Other	4 (6.5)
Type of ICU admission, number (%)	
Medical	33 (53.2)
Surgical	29 (46.8)
Charlson comorbidity score, median (IQR)	2 [0; 4]
Comorbidities, number (%)	
None	22 (35.5)
Diabetes mellitus	24 (38.7)
Chronic heart failure	16 (25.8)
Chronic renal disease	12 (19.4)
Hemodialysis dependent	8 (12.9)
COPD	8 (12.9)
Cirrhosis	2 (3.2)
Creatinine base level, µmol/L, median [IQR]	93 [74; 117]
SAPS II, median [IQR]	56 [40; 73]
SOFA score at Day 0, median [IQR]	8 [6, 11]
Type of infection, number (%)	
Endocarditis of native valve	30 (48.4)
Endocarditis of prosthetic valve	3 (4.8)
Bacteremia without endocarditis	9 (14.5)
Infusion catheter infection	6 (9.7)
Pneumonia	9 (14.5)
Mediastinitis	4 (6.5)
Other	1 (1.6)
Bacteremia, number (%)	56 (90.3)
Weight on D-Day, kg, median [IQR]	88.5 [75.5; 98]
Daily dosing per kg, mg/kg, median [IQR]	105.5 [75; 150]
Plasma concentration, mg/L, median [IQR]	134.3 [65.3; 201]
Serum protein level, g/L	56 [51; 64]
Albuminemia, g/L	20 [13, 21]
Side effects attributed to overdose	
Delirium	8 (12.9)
Persistent coma	3 (4.8)
Acute renal failure	5 (8.1)
SOFA score on D-Day, median [IQR]	8 [6, 13]
Creatinine clearance on D-Day, mL/min	
<10 or hemodialysis	37 (59.7)
10–30	7 (11.3)
>30	18 (29.0)
Type of hemodialysis technique	
CVVHD	4 (12.5)

We defined three different categories of infections: endocarditis (*n* = 33, 53.2%), other endovascular infections (15 patients, 24.2%), and a third group including all the extravascular infections—particularly pneumonia and mediastinitis, representing the remaining 14 patients (22.6%). Bacteremia occurred in fifty-six patients (90.3%). Only two patients underwent extracorporeal life support.

**Fig. 1:**
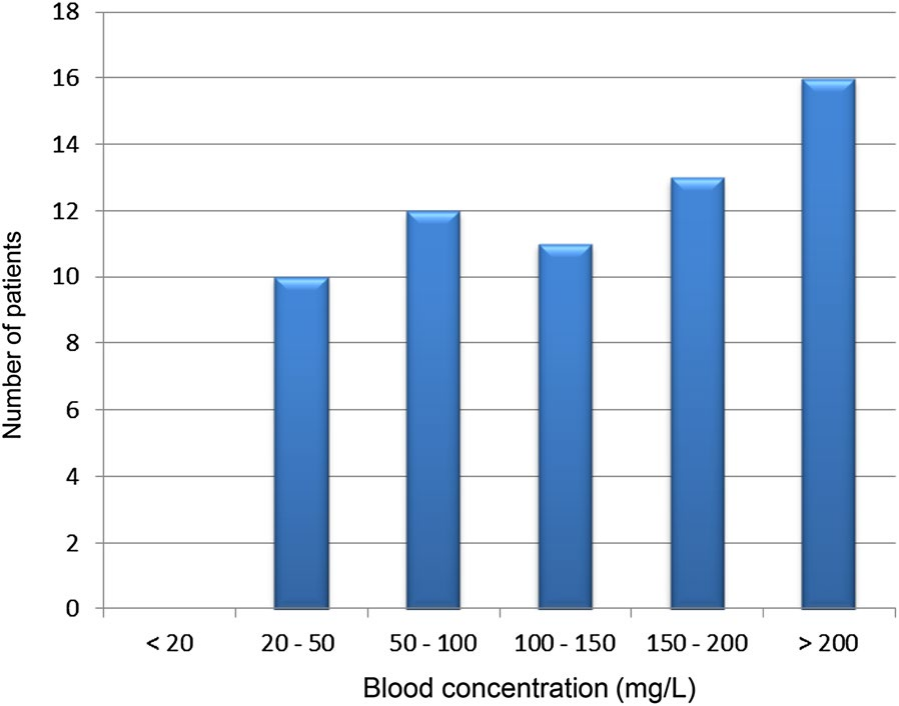
Distribution of antibiotic blood concentrations

Fifty-two patients (83.9%) included in this study had a high plasma concentration in oxacillin or cloxacillin (Fig. 1). Only ten of them reached the trough plasma concentration target of 20 to 50 mg/L. Using a percentage of 10% to estimate the unbound fraction of oxa- or cloxacillin, all patients would have had a trough unbound fraction concentration superior or equal to the maximum MIC for oxa- or cloxacillin sensitivity, i.e. 2 mg/L. As the median ratio albumin/total serum protein level was 33% for the patients with available data, we used the measured or estimated albumin level in our population to estimate the median unbound fraction of oxa- or cloxacillin, which was 18 mg/L.

Twenty-nine patients had at least another antibiotic administered in the last 24 h before dosage: rifampicin (*n* = 14), ofloxacin (*n* = 5), vancomycin (*n* = 4), piperacillin/tazobactam (*n* = 3), clindamycin (*n* = 2), spiramycin (*n* = 1), cefotaxime (*n* = 1), erythromycin (*n* = 1), imipenem (*n* = 1), levofloxacin (*n* = 1), daptomycin (*n* = 1), ciprofloxacin (*n* = 1), and cotrimoxazole (*n* = 1).

**Fig. 2:**
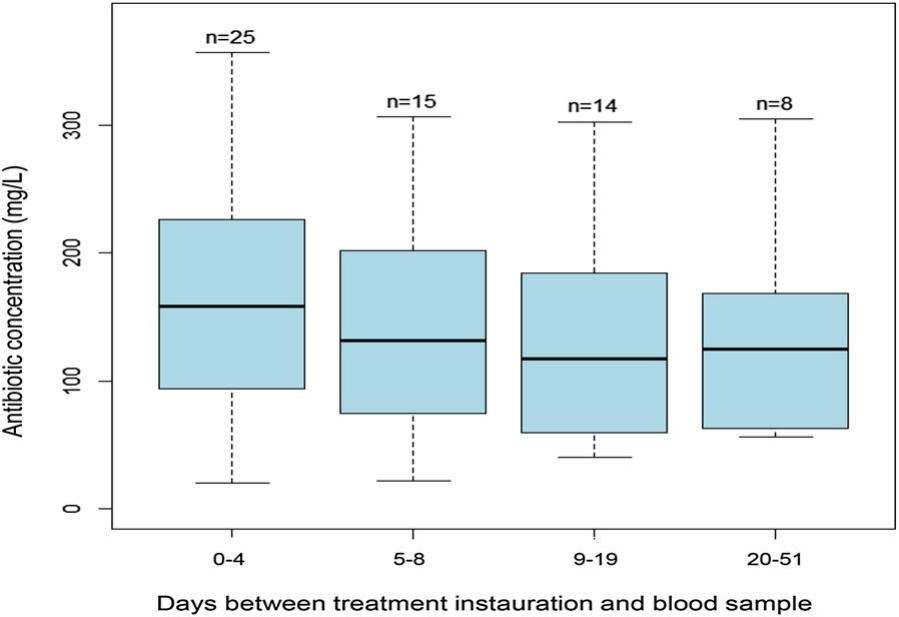
Relationship between antibiotic serum level and day of blood sample

We identified an median time lag of 6 days [4; 11] between the starting date of the treatment and plasma concentration measure. The analysis of trough concentration level on the basis of this time lag did not highlight any variation (Fig. 2). The total dosing of oxa- or cloxacillin was 12 g for 23 patients (37.1%), 8–10 g for 17 patients (27.5%), 6 g or less for 21 patients (33.9%). Only one patient received a dose superior to 12 g, i.e. 16 g. The dose reduction in case of renal failure was statistically significant (Table 2).

**Table 2:** Daily dosing and creatinine clearance

	Creatinine clearance (mL/min)			
	<10	10–30	>30	p*
	*n* = 37	*n* = 7	*n* = 18	
Dosing per 24 h, g, median [IQR]	8 [6–10]	6 [6–12]	12 [10–12]	0.002
Dosing per kg per day, median [IQR]	87.0 [75.0–120.5]	100.0 [52.6–155.8]	131.7 [108.1–160.0]	0.033

* This analysis has been performed with a Mann Whitney test

Through a univariate data analysis on the basis of a logistic regression model, we identified the variables potentially affecting the plasma concentration, i.e. the infected site (endocarditis) and the presence of a bacteremia, the D-Day SOFA score, and a creatinine clearance below 10 mL/min on D-Day. Univariate analysis is shown in detail in Table 3. A multivariate analysis revealed that the variables remaining significant were the dose-to-weight ratio (i.e. the weight on admission, *p* = 0.049) and the creatinine clearance level at the time of plasma concentration measure (*p* = 0.0067).

**Table 3:** Statistical analysis—univariate analysis

	Trough concentration ≤50	Trough concentration >50 mg/L	OR	IC 95%	*p* value
	*n* = 10	*n* = 52	–		–
Age	59.5 [53; 67]	63 [54; 72]	1.026	[0.98; 1.08]	0.29
BMI	26.1 [22.8; 29.1]	28.4 [24.6; 32.8]	1.110	[0.96; 1.29]	0.17
Indication for ICU admission					
Sepsis	4 (40)	34 (65.4)	2.833	[0.71; 11.35]	0.14
Creatinine base level	91 [70; 96]	94 [75; 120]	1.001	[1; 1.01]	0.69
Hemodialysis dependence	1 (10)	7 (13.5)	1.400	[0.15; 12.81]	0.77
SAPS II	52 [36; 73]	56 [40; 71]	1.002	[0.97; 1.04]	0.92
SOFA score on Day 0	7 [4, 10]	9 [6, 11]	1.151	[0.93; 1.42]	0.20
SOFA score on Day 0 without renal failure	7 [3, 9]	7 [5, 8]	1.068	[0.86; 1.33]	0.55
Type of infection					
Infective endocarditis	2 (20)	31 (59.6)	8.611	[1.42; 52.09]	0.02
Other intravascular infection	3 (30)	12 (23.1)	2.222	[0.42; 11.83]	0.35
Other infection	5 (50)	9 (17.3)	1.000		0.06
Bacteremia	7 (70)	49 (94.2)	7.001	[1.17; 41.75]	0.03
Daily dosing per kg, mg/kg	101.3 [75.9; 111.1]	108.1 [75; 150]	1.007	[0.99; 1.02]	0.43
Time lag between treatment initiation and D-Day, d, median [IQR]	5 [3, 7]	6.5 [4; 12.5]	1.099	[0.95; 1.27]	0.19
SOFA on D-Day	7 [4, 8]	9 [6, 14]	1.256	[1.01; 1.56]	0.04
SOFA on D-Day without renal failure	5 [3, 7]	6.5 [4, 10]	1.140	[0.93; 1.4]	0.22
SOFA > 7 on D-Day without renal failure	2 (20)	23 (44.2)	3.172	[0.61; 16.41]	0.17
Creatinine clearance on D-Day, mL/min					
<10 or RRT	2 (20)	35 (67.3)	11.136	[2.01; 61.65]	<0.01
10–30	1 (10)	6 (11.5)	3.818	[0.38; 38.83]	0.26
>30	7 (70)	11 (21.2)	1.000		0.02
Aminoglycosides	4 (40)	27 (51.9)	1.620	[0.41; 6.42]	0.49
Serum protein level on D-Day, g/L			0.944	[0.88; 1.01]	0.11

Fourteen patients (22.6%) experienced side effects attributed to an overdosing in oxa- or cloxacillin, as measured by the microbiological method (i.e. representing the total antimicrobial activity in plasma). Eleven (17.7%) presented with neurological symptoms, i.e. persistent coma for three patients and delirium for eight patients (12.9%). The median trough concentration of the antibiotic for those patients was 165 mg/L, with a minimum at 97 mg/L, and a maximum at 302 mg/L. Head CT scans showed no abnormalities in four patients, and formerly known strokes for 5. An electroencephalogram (EEG) was performed for six of the 11 patients and showed the presence of reactivity but a slow background activity. Taking into account the other medications administered to patients (i.e. sedatives and other antibiotics), the Naranjo score for the six patients with an EEG was 6, and 5 for the five other ones, which classified the observed neurological events as ‘probably’ due to oxacillin overdosing. Comparing the patients with and without neurological side effects, we found no statistically significant differences in terms of age, weight, renal function, length of stay (respectively, 64 vs 42 days, *p* = 0.14), or mortality at day 90 (41.7 vs 52%, *p* = 0.52), but the median duration of treatment was longer (32.5 vs 14 days, *p* = 0.03). Neurological evolution was favorable after reduction in treatment dose in all patients.

## Discussion

Our study is the first one to thoroughly evaluate oxacillin dosage in the context of extremely severe infections. We found a plasma trough concentration of oxa- or cloxacillin above the target level for a large proportion of patients and a quarter of them experienced side effects that were attributed to the high antibiotic concentration. The latest international recommendations for the treatment of endocarditis provide for a cloxacillin or oxacillin dose of 12 g/j for an adult, without reference to weight or renal function [5]. It is interesting to note that these recommendations have been slightly modified in the past 40 years, although the relevant patients population has changed; the average age of patients, in particular, has tremendously increased and concomitantly the proportion of patients with impaired renal function [26]. In the literature, the data related to a modification of the dosing of these antibiotics in case of impaired renal function are weak and contradictory. In the book ‘The use of antibiotics’ by Kucer, it is mentioned that the dose should normally be reduced in case of impaired renal function, and especially if very high intravenous doses are used [27]. Other recommendations point in the same direction, notably from the HAS in 2012, whereby a reduction in the doses by half is proposed in case of renal impairment and creatinine clearance below 30 mL/min [12]. However, none of these recommendations rely on pharmokinetics data published in the literature. There are very few differences between oxa- and cloxacillin PK/PD parameters [27]. As said before, both are highly bound to albumin, i.e. more than 90%, and distribution of the two drugs in the body after parenteral administration is similar. After IV administration, 70–80% of the injected dose of cloxacillin is eliminated by urinary route in 6 h, in an active form, and the rest by the biliary tract, also in active form; the biliary elimination is more marked for oxacillin. No data are available in the literature regarding potential differences between their metabolites’ toxicity. A first pharmacological study published by Nauta et al. [28] assessed pharmacokinetics of orally administered cloxacillin in 11 healthy subjects and 11 patients on chronic intermittent hemodialysis. They found a cloxacillin half-life (*T*_1/2_) 3 times higher in hemodialysis patients than in healthy ones, but a lower absorption. Thus, they recommended using the same dosing in anuric patients, but this did not concern intravenous administration. Thijssen and Wolters [29] observed a prolonged half-life of antibacterial activity in plasma of patients treated with flucloxacillin and presenting with renal failure, mainly because of the accumulation of an active metabolite, hydroxyflucloxacillin. This metabolite has a longer half-life than flucloxacillin, and a lower plasma clearance by a non-renal route. Although some accumulation of the hydroxyflucloxacillin metabolite was observed, they recommended no adjustment of the flucloxacillin dose in severe renal impairment, because of a low metabolite toxicity.

All of these data did not concern ICU patients. PK/PD of beta-lactams in an intensive care context is very complex and often unpredictable [8]. Although an increase in cardiac output, glomerular filtration, and distribution volume would make the case for a higher dosing, the emergence of an acute renal failure exposes the patient to the risk of an overdosing [30]. Moreover, the target to reach a 100% *T* > MIC often leads to increase treatment doses.

Verdier et al. [31] analyzed plasma concentrations of oxacillin in 57 patients, half of them being treated for endocarditis. Twenty-nine patients had an altered creatinine clearance below 60 mL/min, as estimated with the Cockcroft and Gault formula, but very few of them had a clearance of less than 10 mL/min; furthermore, no renal replacement therapy was mentioned. However, they showed that plasma concentrations were higher in case of altered renal function and highlighted the interindividual variability of concentrations after administration of high doses of cloxacillin. Visser et al. [32] suggested the existence of variations of a saturable mechanism of tubular excretion, explaining the accumulation of antibiotics during treatments with continuous infusions even in patients with normal renal function. In this latter study, the authors tried to predict plasma concentration of cloxacillin administered in continuous infusions with an equation including creatinine clearance and weight; patients with a creatinine clearance inferior to 10 mL/min had to be excluded of the study, because the prediction remained unreliable.

 Several studies reported neurological side effects of these molecules [33, 34]. Malone et al. [13] reported convulsions with a concentration of 270 mg/L. Delirium increases the duration of ICU stay and mechanical ventilation and is associated with a poor outcome, including subsequent cognitive decline and mortality [35, 36]. We found in our study 17.7% of patients suffering from neurological adverse effects that were attributed by the clinician in charge to an overdosing in oxa- or cloxacillin. Patients were receiving several medications, including sedatives, at the same time, and retrospective design makes it difficult to establish the responsibility of the overdosing. Nevertheless, other frequent causes of delirium as pain, new cerebral lesions or sepsis were ruled out, and neurological recovery was observed after stopping or adapting treatment dose. The Naranjo scores classified those adverse events as ‘probably’ due to the antibiotic.

Considering the high proportion of patients with renal replacement therapy included in our study, it appears that this population is particularly at risk of overdosing and must be accurately monitored.

Our study had several limitations. Data were collected retrospectively for 25 patients (40.3%) before 2012, but there was no difference in the dosage of administered antibiotics between the two study periods. Moreover, the day retained for collecting the blood samples was not the same for all patients; even if the dosing was made prospectively after 2012, blood samples could only be collected and analyzed in the laboratory on week days. Nonetheless, statistical analysis did not reveal any difference in the median trough concentration based upon the day of the measure. Finally, before 2012, the reason for assessing oxa- or cloxacillin concentration was not mentioned; we could suppose clinicians suspected an overdosing because of observed side effects. However, among those 25 patients, only three were reported to have experienced adverse effects due to overdosing.

It has also to be noted that the oxa- and cloxacillin assay used in our study represents a total antibiotic activity of oxa- and cloxacillin, thus including all active metabolites, but does not evaluate its unbound fraction. This method is the same as the one used by Nauta and Mattie [37] and Bulger et al. [38] in former studies. Verdier et al. [31] used high-performance liquid chromatography (HPLC) to measure antibiotic concentrations that could explain their lower proportion of overdosing in normo-renal patients. Whether we should take into account the presence of the active metabolites or not to determine the optimal trough concentration target to reach is not known. Indeed, it is interesting to notice that the MIC of oxacillin or cloxacillin metabolites for *Staphylococcus aureus* is equivalent to that of the parent compounds, i.e. 0.5–1.2 mg/L, in the study by Thijssen and Mattie [39]. In the same study, they observed that the amount of active metabolites represented 40–50% of the plasmatic penicillin activity in patients on chronic intermittent hemodialysis. Thus, it appears that the microbiological method may more accurately estimate the total antimicrobial activity in the plasma. Whether or not the metabolites are as effective as the parent compound to treat infections in vivo is not known, since their distribution may be different. However, in the case of endocarditis or endovascular infections, this might not influence the outcome of therapy.

As compared to microbiological method, HPLC dosing method has also the advantage to give clinicians a result more rapidly. However, in our center, we were able to obtain a trough concentration of oxa- or cloxacillin in 48 h, which was considered to be a reasonable delay to adapt treatment dosing.

Due to the retrospective design of our study, we were not able to measure the unbound fraction of oxa- or cloxacillin in our patients’ plasma. Ulldemolins et al. [25] showed that the unbound fraction of flucloxacillin was more important in case of hypoalbuminemia, leading to an increased volume of distribution of the molecule and to a risk of underdosing. As most of our patients had a low serum protein level and consequently a low albumin concentration, we estimated that the unbound fraction of oxa- or cloxacillin was also increased, compared to healthy patients in which it represents 10% of the total antibiotic concentration. If we extrapolate the unbound fraction increase in the same proportion of the decrease in albumin concentration, none of our patients would have had an unbound oxa- or cloxacillin trough level inferior to 2 mg/L. Considering the very high concentrations obtained in our population with an important proportion of patients with impaired renal function, the role of albumin in pharmacokinetics and pharmacodynamics of highly protein-bound antibiotics, like oxacillin, might be of less relevance. As suggested by Benet and Hoener [40], changes in plasma protein binding will usually not influence the clinical exposure of a patient to a drug, except for some specific cases of a drug with a high extraction ratio and narrow therapeutic index that is intravenously administered. Moreover, the study by Ulldemolins et al. did not consider the plasma concentration of non-protein-bound fraction of hydroxyflucloxacillin, which may play a part in antimicrobial efficacy, and which is estimated to be twice as large as that of its parent compound in previous studies [29].

We therefore deem it essential to monitor and adapt dosing of frequently used molecules in intensive care, including antibiotics. In order to avoid antibiotic accumulation in our ICU patients, a systematic plasma concentration measure of oxacillin has been set in our unit. The efficiency of this provision has to be determined.

Through this study, we could identify the need for renal replacement therapy as a major risk factor for overdosing. In spite of a reduction in dosage in case of renal failure, it did not allow us to avoid overdosing in our intensive care population. Even if the severity of our patients’ condition may be an additional risk factor for overdosing in an ICU context, the proposed reduction by half of the dosage when the creatinine clearance is less than 30 mL/min is far from satisfactory. Further pharmacokinetic study is needed to clarify the mechanisms of this overdosing and to provide guidelines for dosage when facing renal function impairment.

We therefore propose to develop a systematic therapeutic drug monitoring for oxa- and cloxacillin in ICU, as could be done for aminoglycosides or vancomycin, in order to optimize the managing and monitoring of patients. Faster techniques such as HPLC have largely developed these last few years, allowing for a reliable plasma concentration measure of numerous molecules [41].

Nevertheless, the question concerning the efficiency of this dosing method to evaluate the accumulation of active metabolites and their potential toxicity remains unanswered.

## Conclusions

With the proposed reduction by half of the dosage of oxa- and cloxacillin when the creatinine clearance is less than 30 mL/min, a large proportion of patients remain overdosed. In multivariate analysis, identified risk factors for overdosing were the dose-to-weight ratio and an impaired renal function. Considering the severity of the observed side effects, especially coma and delirium, ICU patients with renal failure must be accurately monitored.

## Additional file

**Additional file 1: Figure S1.** Flowchart of patients included between 2012 and 2014.

## Abbreviations

ICU: intensive care unit
IQR: interquartile range
MSSA: methicillin-susceptible *Staphylococcus aureus*
MIC: minimum inhibitory concentration
HAS: Haute Autorité de Santé (French health authority)
SOFA: Sequential Organ Failure Assessment
SAPS: Simplified Acute Physiology Score
CEERB: Comité d’Evaluation de l’Ethique des projets de Recherche Biomédicale Paris Nord
CNIL: Commission Nationale de l’Informatique et des Libertés
BMI: body mass index
EEG: ElectroEncephaloGram
EUCAST: European Committee on Antimicrobial Susceptibility Testing (EUCAST)
HPLC: high-performance liquid chromatography
