# Supplementary material for: Systematic overdosing of oxa- and cloxacillin in severe infections treated in ICU: risk factors and side effects
Source: Ann Intensive Care. 2017 Mar 22;7:34. doi: 10.1186/s13613-017-0255-8 (PMC5362565; doi:10.1186/s13613-017-0255-8)
Supplement: Supplementary file 1 — Additional file 1: Figure S1. Flowchart of patients included between 2012 and 2014. [file 13613_2017_255_MOESM1_ESM.docx]

Figure S1. Flow charts of patients between 2012 and 2014

102 patients receiving oxa- or cloxacillin in ICU between 2012 and january 2014

53 patients receiving oxa- or cloxacllin in ICU for more than 72h

4 patients died before 72h of treatment

18 patients had a treatment stopped or modified before 72h

22 were discharged of ICU before 72h

5 patients were excluded because of decision for palliative care

16 patients were excluded because of missing data

37 patients analyzed
